# Neither load nor systemic hormones determine resistance training-mediated hypertrophy or strength gains in resistance-trained young men

**DOI:** 10.1152/japplphysiol.00154.2016

**Published:** 2016-05-12

**Authors:** Robert W. Morton, Sara Y. Oikawa, Christopher G. Wavell, Nicole Mazara, Chris McGlory, Joe Quadrilatero, Brittany L. Baechler, Steven K. Baker, Stuart M. Phillips

**Affiliations:** ^1^Department of Kinesiology, McMaster University, Hamilton, Ontario, Canada;; ^2^Department of Kinesiology, University of Waterloo, Waterloo, Ontario, Canada; and; ^3^Department of Neurology, School of Medicine, McMaster University, Hamilton, Ontario, Canada

**Keywords:** load, testosterone, growth hormone, anabolism, strength training

## Abstract

We reported, using a unilateral resistance training (RT) model, that training with high or low loads (mass per repetition) resulted in similar muscle hypertrophy and strength improvements in RT-naïve subjects. Here we aimed to determine whether the same was true in men with previous RT experience using a whole-body RT program and whether postexercise systemic hormone concentrations were related to changes in hypertrophy and strength. Forty-nine resistance-trained men (23 ± 1 yr, mean ± SE) performed 12 wk of whole-body RT. Subjects were randomly allocated into a higher-repetition (HR) group who lifted loads of ∼30-50% of their maximal strength (1RM) for 20–25 repetitions/set (*n* = 24) or a lower-repetition (LR) group (∼75–90% 1RM, 8–12 repetitions/set, *n* = 25), with all sets being performed to volitional failure. Skeletal muscle biopsies, strength testing, dual-energy X-ray absorptiometry scans, and acute changes in systemic hormone concentrations were examined pretraining and posttraining. In response to RT, 1RM strength increased for all exercises in both groups (*P* < 0.01), with only the change in bench press being significantly different between groups (HR, 9 ± 1, vs. LR, 14 ± 1 kg, *P* = 0.012). Fat- and bone-free (lean) body mass and type I and type II muscle fiber cross-sectional area increased following training (*P* < 0.01) with no significant differences between groups. No significant correlations between the acute postexercise rise in any purported anabolic hormone and the change in strength or hypertrophy were found. In congruence with our previous work, acute postexercise systemic hormonal rises are not related to or in any way indicative of RT-mediated gains in muscle mass or strength. Our data show that in resistance-trained individuals, load, when exercises are performed to volitional failure, does not dictate hypertrophy or, for the most part, strength gains.

## Abstract (precis)

*We provide novel evidence of the effect of lifting markedly different (lighter vs. heavier) loads (mass per repetition) during whole-body resistance training on the development of muscle strength and hypertrophy in previously trained persons. Using a large sample size (n = 49), and contradicting dogma, we report that the relative load lifted per repetition does not determine skeletal muscle hypertrophy or, for the most part, strength development. In line with our previous work, acute postexercise systemic hormonal changes were unrelated to strength and hypertrophic gains*.

## NEW & NOTEWORTHY

*We provide novel evidence of the effect of lifting markedly different (lighter vs. heavier) loads (mass per repetition) during whole-body resistance training on the development of muscle strength and hypertrophy in previously trained persons. Using a large sample size (n = 49), and contradicting dogma, we report that the relative load lifted per repetition does not determine skeletal muscle hypertrophy or, for the most part, strength development. In line with our previous work, acute postexercise systemic hormonal changes were unrelated to strength and hypertrophic gains*.

resistance training (RT) is a potent stimulus for increasing skeletal muscle mass and strength (9, 30); however, the exact RT variables that determine skeletal muscle hypertrophy and strength remain a topic of continued investigation (3, 36). Current recommendations are that RT with relatively heavy [i.e., at ∼70–85% one-repetition maximum (1RM)] loads (“load” herein referring to the amount of mass used per repetition) is a prerequisite for maximizing RT-induced hypertrophy (12, 31). It has even been suggested, on the basis of only acute electromyography (EMG) data [despite caution on use of EMG in this manner (10)], that greater motor unit recruitment occurs when lifting heavier loads even if heavier and lighter loads are performed to volitional failure (16, 21). Notably, this conclusion is at odds with existing data determined from long-term training studies (28, 33). We reported that load from as low as 30% and up to 90% of 1RM played a minimal role in stimulating muscle protein synthesis (4). Similar loading strategies also did not affect hypertrophy in a small sample of trained (33) or untrained (28) men following RT when the participants performed their RT to volitional failure. In addition, and in contrast to what others have proposed (18, 19, 31), we have also demonstrated that resistance exercise-induced increases in circulating hormones play little role in regulating muscle protein synthesis after an acute bout of resistance exercise (51) or skeletal muscle hypertrophy following RT (50). Taken together, our data suggest that factors regulating skeletal muscle hypertrophy in response to RT include neither load nor systemic hormonal concentrations (4, 28, 33, 50, 51).

While there is growing evidence that neither load (28, 33) nor acute postexercise increases in circulating hormones (50) affect RT-induced skeletal muscle hypertrophy, it is important to acknowledge that many of the aforementioned studies were conducted in healthy, but untrained participants (4, 28, 50, 51). Given that resistance-trained individuals exhibit an attenuated muscle protein synthetic response to resistance exercise (17, 53), they are likely less “adaptable” than untrained persons in terms of phenotypic adaptations of skeletal muscle in response to RT. In addition, the model used in previous trials (4, 28) was unilateral in nature, which is not a training model used in practice, and limb cross-education may have obscured a true estimate of strength development with the comparison of lighter vs. heavier loads (6).

The primary aim of this study was to determine the effects of a 12-wk higher-repetition (lower load) vs. a lower-repetition (higher load) RT intervention on skeletal muscle hypertrophy and strength development in resistance-trained young men. The secondary aim was to examine whether the acute postexercise increase in systemic hormones was correlated with changes in skeletal muscle mass or strength. Our hypothesis was that neither load nor the acute postexercise increase in systemic hormones would determine RT-induced adaptations.

## METHODS

### 

#### Participants.

Forty-nine healthy young men (23 ± 1 yr, 86 ± 2 kg, 181 ± 1 cm, means ± SE) who had been engaging in RT for at least the past 2 yr [4 ± 2 yr, training >2 sessions per week (range 3–6 days/wk), including at least one weekly dedicated lower body session] volunteered to participate in this study. Recognizing the high interindividual response variability in hypertrophy and strength gain that occurs with RT (13, 27, 28, 48), we conducted the study with a large enough number of participants to allow detection of a 15% difference in hypertrophy via muscle fiber cross-sectional area (CSA) change and a 10% difference in fat- and bone-free (lean) body mass change measured by dual-energy X-ray absorptiometry (DXA) with 90% power based on previous work in trained men (33).

#### Ethics statement.

All participants were informed of the purpose of the study, experimental procedures, and associated risks prior to participation and exercise testing. All participants gave verbal and written informed consent, which was approved by the Hamilton Integrated Research Ethics Board and conformed to the most recent Tri-Council policy statement on the use of human participants in research (http://www.pre.ethics.gc.ca/pdf/eng/tcps2-2014/TCPS_2_FINAL_Web.pdf). The trial was registered at https://clinicaltrials.gov as NCT02139865.

#### Familiarization and strength testing.

Two weeks prior to the start of the RT protocol, participants completed a familiarization session to assess each participant's 10RM for each exercise. At least 72 h after any exercise, participants returned to the laboratory to complete 1RM (strength) testing on the inclined leg press (LP; Maxam Fitness, Hamilton, ON, Canada), barbell bench press (BP), machine-guided knee extension (KE; Atlantis, Laval, QC, Canada), and machine-guided shoulder press (SP; Life Fitness, Rosemont, IL). The same investigators administered all strength testing. In short, after a brief general warm-up, a specific warm-up of the given exercise was then performed at ∼50% of the participant's estimated 1RM based on the 10RM testing. Load was progressively increased by ∼10–20% for each repetition until a true 1RM was reached as previously described (5, 40). Three to five minutes of rest was given between each attempt. A successful attempt required the participant to move the load throughout the full range of motion with correct form.

#### Experimental design.

**Fig. 1.**
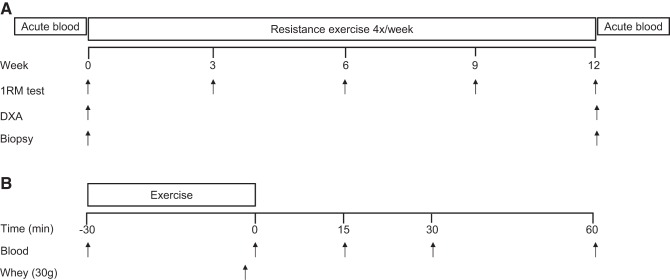
Schematic representation of study protocol (*A*) and acute blood sampling protocol (*B*).

**Fig. 2.**
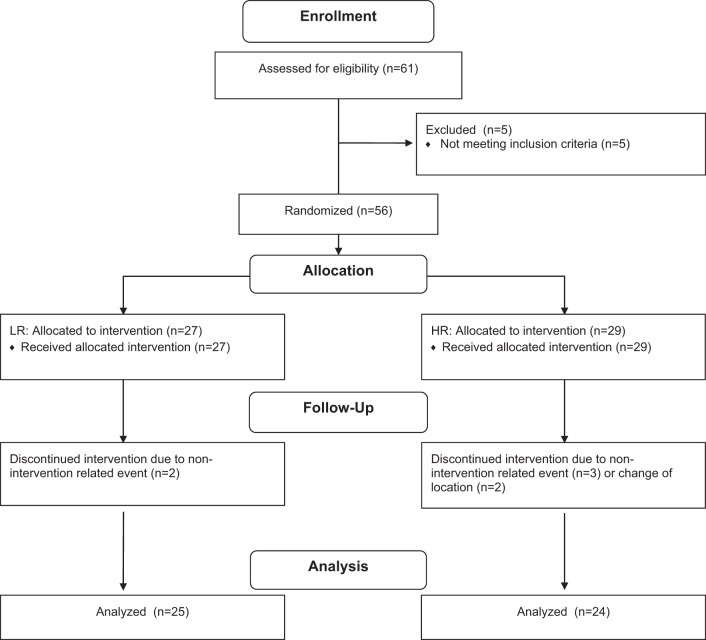
Group allocation.

A schematic illustration of the experimental design can be seen in Fig. 1*A*. A between-group, repeated measures design in which participants were randomly allocated to one of two possible conditions, high repetition (HR; *n* = 29) or low repetition (LR; *n* = 27; Fig. 2), was employed. For the training program the HR group performed 3 sets of 20–25 repetitions per set such that the load varied between ∼30 and 50% of 1RM with each set being performed to volitional failure. The LR group performed 3 sets of 8–12 repetitions per set that corresponded to ∼75–90% of 1RM with each set being performed to volitional failure (38). The loads were adjusted in between each set to ensure that the correct repetition range was maintained. Each participant underwent 12 wk of full-body RT 4 days per week. Session attendance was 97 ± 2% for the HR group and 96 ± 2% for the LR group with no difference between groups. Both groups performed 1RM testing at baseline and retested at 3, 6, 9, and 12 wk on what would be the participants' first session of the week. Participants consumed 30 g of whey protein (BioPRO; Davisco Foods International, Le Sueur, MN) twice per day: immediately following RT on training days (8) and the other prior to sleep (39). On nontraining days, participants consumed the first dose in the morning and the second dose 1–2 h prior to sleep, similar to training days.

#### Acute protocol.

A schematic illustration of the acute blood sampling protocol can be seen in Fig. 1*B*. At least 72 h following the familiarization and strength testing, each participant came in after an overnight fast and received a muscle biopsy from the vastus lateralis and a resting blood sample via an intravenous antecubital cannula. Following the resting blood draw, a bout of resistance exercise was performed that consisted of a “superset” (exercises conducted in succession with no rest in between) including an incline leg press, hamstring curl, and knee extension. Participants were given 1 min of rest following each superset with three supersets performed in total. Each exercise was performed until volitional failure in their respective group repetition ranges (HR or LR). Following the bout of resistance exercise, the participant was given 30 g of pure whey protein (BioPRO; Davisco Foods International) mixed with 500 ml of water. Blood samples were collected at 0 (immediately post), 15, 30, and 60 min following the consumption of the protein beverage.

#### Hormone concentrations.

Blood samples were obtained via a cannula that was inserted into an antecubital vein kept patent by periodic flushes of 0.9% saline. Tubes containing whole blood were allowed to clot for 30 min at room temperature before serum (4 ml) was isolated. Heparinized tubes were used to isolate plasma (4 ml). All blood tubes were centrifuged at 4,000 *g* for 10 min at 4°C prior to serum and plasma being separated into cryotubes and frozen at −80°C until further analysis. Blood samples were analyzed for serum total testosterone (T; ng/dl), free T (fT; pg/ml), cortisol (nM), dihydrotestosterone (DHT; ng/ml), dehydroepiandrosterone (DHEA; ng/ml), luteinizing hormone (LH; IU/l), insulin-like growth factor 1 (IGF-1; μg/dl), free IGF-1 (fIGF-1; ng/ml), lactate (mM), and growth hormone (GH; ng/ml) using solid-phase, two-site chemiluminescence immunometric assays (Immulite; Intermedico, Holliston, MA) or radio-immunoassay (Diagnostics Products, Los Angeles, CA). All analyses resulted in interassay coefficients of variation (CV; *n* = 245) of less than 6% and intraassay CV (*n* = 2,450) on replicates of less than 4%.

#### Body composition.

Body composition was assessed following an overnight fast (12 h) and >72 h following their last exercise bout both preintervention and postintervention. DXA measurements were conducted using a GE Lunar iDXA total body scanner (GE Medical Systems Lunar, Madison, WI) and analyzed with software (Lunar enCORE version 14.1; GE Medical Systems Lunar) in the medium scan mode. The machine was calibrated each testing day by using a three-compartment Universal Whole Body DXA Phantom: Oscar, Jr (Orthometrix, Naples, FL). The analysis regions used were standard regions where the head, torso, arms, and legs were subdivided by the software, but were subsequently checked manually, in a blinded manner, by a single investigator. Intrascan (without repositioning) and interscan (on different occasions) variability using the phantom was <1.6% for all tissues.

#### Dietary records.

Dietary intake records were collected at 0, 3, 6, 9, and 12 wk and analyzed using the NutriBase dietary analysis software (Nutribase11 Professional Edition, version 11.5; Cybersoft, Phoenix, AZ).

#### Resistance-training intervention.

The full-body RT was performed 4 days/wk (Monday, Tuesday, Thursday, and Friday). Each day included five exercises, consisting of two separate supersets and one additional exercise. Exercises were performed for three sets, with each set executed until volitional failure. One minute of rest was given between each set or superset. Each workout was repeated twice per week [Monday/Thursday: inclined leg press with seated row (superset 1), barbell bench press with cable hamstring curl (superset 2), and front planks (set 3). Tuesday/Friday: machine-guided shoulder press with bicep curls (superset 1), triceps extension with wide-grip pull downs (superset 2), and machine-guided knee extension (set 3)]. If necessary, loads were decreased (∼5–10%) between sets to ensure repetitions were performed within the participant's assigned repetition range. Each participant was individually supervised by a trainer for each session to ensure each set was performed to volitional failure with correct technique. Participants' load was increased with subsequent training sessions when they could perform more repetitions than their designated repetition range. Weeks during the training intervention that included 1RM testing (*weeks 4*, *7*, and *10*) involved only three prescribed sessions with 1RM testing to serve as the fourth session. Participants were asked to refrain from any additional exercise outside of the study.

#### Volume.

The volume, sometimes referred to as “volume-load,” of each set was calculated by multiplying the number of repetitions with the load. Total volume was calculated as the sum of each set's volume throughout the 12-wk RT intervention. Average session volume was calculated by dividing the total volume by the number of sessions that participant attended.

#### Muscle fiber type and cross-sectional area.

Muscle biopsies were obtained from the vastus lateralis preintervention and postintervention. Biopsies were taken using a 5-mm Bergström needle custom modified for manual suction under local anesthesia (1% lidocaine). Participants had not participated in any physical activity for 72 h prior to each biopsy. Upon excision, the muscle samples were immediately cleared of visible connective tissue and fat and were oriented vertically by visual inspection before being embedded in optimal cutting temperature medium. The mounted muscle was frozen in isopentane, cooled by liquid nitrogen, and stored at −80°C until further analysis. Cross sections (7-μm thick) were cut on a Microm HM550 Cyrostat (Thermo Fisher Scientific, Waltham, MA), mounted on glass slides, and stained. Fiber type and CSA were assessed via immunofluorescent staining of myosin heavy chain (MHC) isoforms and dystrophin as previously described (2, 37). Primary antibodies against dystrophin (MANDYs), MHCI (BA-F8), MHCIIA (SC-71), and MHCIIX (6H1; Developmental Studies Hybridoma Bank, Iowa City, IA) followed by isotope-specific fluorescent secondary antibodies allowed for the identification of type I, type IIA, and type IIX fibers. Slides were mounted with Prolong Diamond Antifade Reagent (Life Technologies, Burlington, ON, Canada) and imaged the following day. Images were taken with a Nikon Eclipse 90i microscope at a magnification of 20X and captured with a Photometrics Cool SNAP HQ2 fluorescent camera (Nikon Instruments, Melville, NY). Analysis was completed using the Nikon NIS elements AR software (Nikon Instruments) on a large-scale image. All data reported in this manuscript, unless otherwise stated, have type IIA and type IIX fiber types pooled together and reported as type II fibers because of the number necessary to individually analyze type IIA and IIX fibers (∼50–60) per sample (24, 25). Fiber CSA was determined by counting at least 100 individual fibers, and fiber type was assessed using the whole cross section of fibers (367 ± 18 fibers). All fibers selected for analysis were free of freezing artifact, and care was taken so that obliquely or longitudinally oriented fibers were not used in the analysis. Muscle fibers on the periphery of muscle cross sections were not used in the analysis. The same investigator, who was blinded to the time and group of each sample, conducted all immunofluorescent analyses. All mention of CSA refers to the muscle fiber CSA determined by muscle biopsy.

#### Statistical analysis.

All analyses were performed using SPSS (version 22.0; Chicago, IL). Baseline characteristics were compared between groups using an independent *t*-test. The postexercise hormonal area under the curve (AUC) was calculated by subtracting the baseline concentration from the postexercise AUC of each hormone (60 min). Bivariate correlations were run for the two-tailed Pearson correlation coefficient between the postexercise hormone AUC and the change in strength and muscle mass. Muscle strength, lean body mass, muscle fiber CSA, muscle fiber type, and postexercise hormonal AUC were all analyzed using a two-factor (group × time) repeated measures analysis of variance (ANOVA) with group (between) and time (within) as the experimental variables. In addition, independent *t*-tests were performed with the independent variable as condition and the dependent variable as the absolute change for each measure of strength and muscle mass, all reported with their mean and 95% confidence intervals (CI). Statistical significance was accepted when *P* ≤ 0.05. Results are presented as means ± SE in text and tables unless otherwise specified. To show the variability in response, graphs are presented as box-and-whisker plots including the median (lines), mean (crosses), interquartile range (boxes), and 95% CI (tails).

## RESULTS

### 

#### Descriptive characteristics.

Forty-nine participants completed this study (Table 1). Participants were similar at baseline for all descriptive characteristics with no differences between groups (*P* > 0.05) with the exception of fat mass (*P* < 0.05; Table 1). Seven participants did not complete the study protocol because of non-intervention-related injuries (*n* = 5) or relocation (*n* = 2; Fig. 2). There was no significant difference in dietary intake of macronutrients or energy between groups at 0, 3, 6, 9, or 12 wk (*P* > 0.05; data not shown).

**Table 1.** Participants' baseline characteristics

	HR (*n* = 24)	LR (*n* = 25)	*P*
Age, yr	23 ± 2	23 ± 2	0.73
Training age, yr	4.2 ± 2	4.6 ± 3	0.54
Total body mass, kg	88 ± 4	85 ± 2	0.57
Height, m	1.81 ± 1	1.80 ± 1	0.81
BMI, kg/m^2^	26.9 ± 2	26.0 ± 2	0.41
Lean mass, kg	65.7 ± 2	65.7 ± 1	0.99
Total fat mass, kg	19.4 ± 2	16.9 ± 1	0.03
Leg press 1RM, kg	357 ± 21	353 ± 13	0.87
Bench press 1RM, kg	98 ± 4	97 ± 4	0.88
Knee extension 1RM, kg	76 ± 3	76 ± 3	0.92
Shoulder press 1RM, kg	91 ± 5	92 ± 4	0.87

Values are means ± SE. BMI, body mass index.

#### Body composition and muscle fiber CSA.

**Fig. 3.**
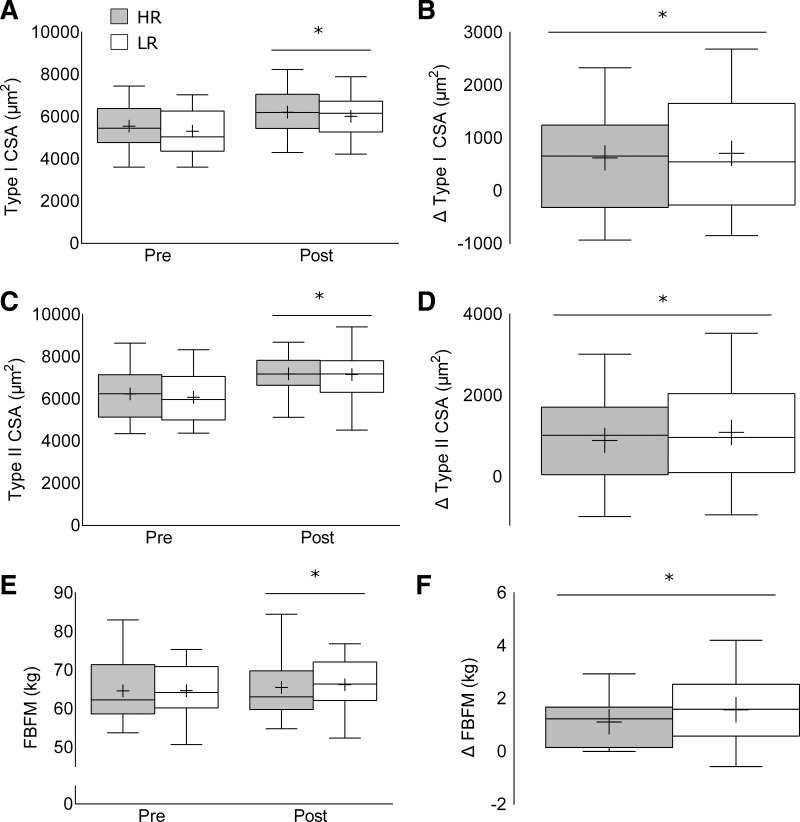
Fiber cross-sectional area (CSA) and body composition changes in the high-repetition (HR) and low-repetition (LR) groups following 12 wk of resistance training including type I CSA absolute values (*A*) and change following training (*B*), type II fiber CSA absolute values (*C*) and change following training (*D*), and fat- and bone-free (lean) body mass (FBFM) absolute values (*E*) and change following training (*F*). Values are presented as median (lines) with interquartile range (boxes) ± range (minimum and maximum), where + indicates mean. *Significantly different (*P* < 0.05) from baseline.

Kolmogorov-Smirnov and Levene's tests were run for normality and homogeneity of variance, respectively, and all assumptions were met (*P* > 0.05). Following the intervention (using pooled means), there was an increase in type I [5,448 ± 152 to 6,113 ± 150 μm^2^; *F*(1,47) = 19.45, *P* < 0.001; Fig. 3*B*] and type II [6,193 ± 176 to 7,171 ± 158 μm^2^; *F*(1,47) = 26.11, *P* < 0.001; Fig. 3*D*] CSA with no significant difference between groups. Independent *t*-tests on the absolute change also revealed no difference between groups for muscle fiber CSA in either type I [*t*(47) = −0.29, *P* = 0.77, mean (M) = −88, 95% CI (−693, 518)] or type II [*t*(47) = −0.52, *P* = 0.61, M = −198, 95% CI (−967, 569)].

There were no group, time, or group by time interactions for type I and type II fiber type distributions with the intervention; however, with means pooled and all fiber types included (type I, IIA, and IIX), there was a shift from type IIX [10.3 ± 1.1 to 6.5 ± 0.72%; *F*(1,47) = 8.95, *P* = 0.004] to type IIA fibers [45 ± 1.7 to 49.7 ± 1.2%; *F*(1,47) = 5.11, *P* = 0.03].

Following the intervention (using pooled means), there was a significant increase in total fat- and bone-free mass [FBFM; 64.6 ± 1.1 to 65.8 ± 1.1 kg; *F*(1,47) = 40.50, *P* < 0.01; Fig. 3*F*] with no significant difference between groups indicated by ANOVA and by an independent *t*-test [*t*(47) = −1.91, *P* = 0.091, M = −0.73, 95% CI (−1.49, 0.04)]. There was also a significant increase in appendicular lean mass [ALM; 33.1 ± 0.6 to 34.0 ± 0.6 kg; *F*(1,47) = 30.19, *P* < 0.001] and leg lean mass [LLM; 24.4 ± 0.5 to 25.0 ± 0.5 kg; *F*(1,47) = 16.97, *P* < 0.001] with no significant differences between groups.

#### Strength.

**Fig. 4.**
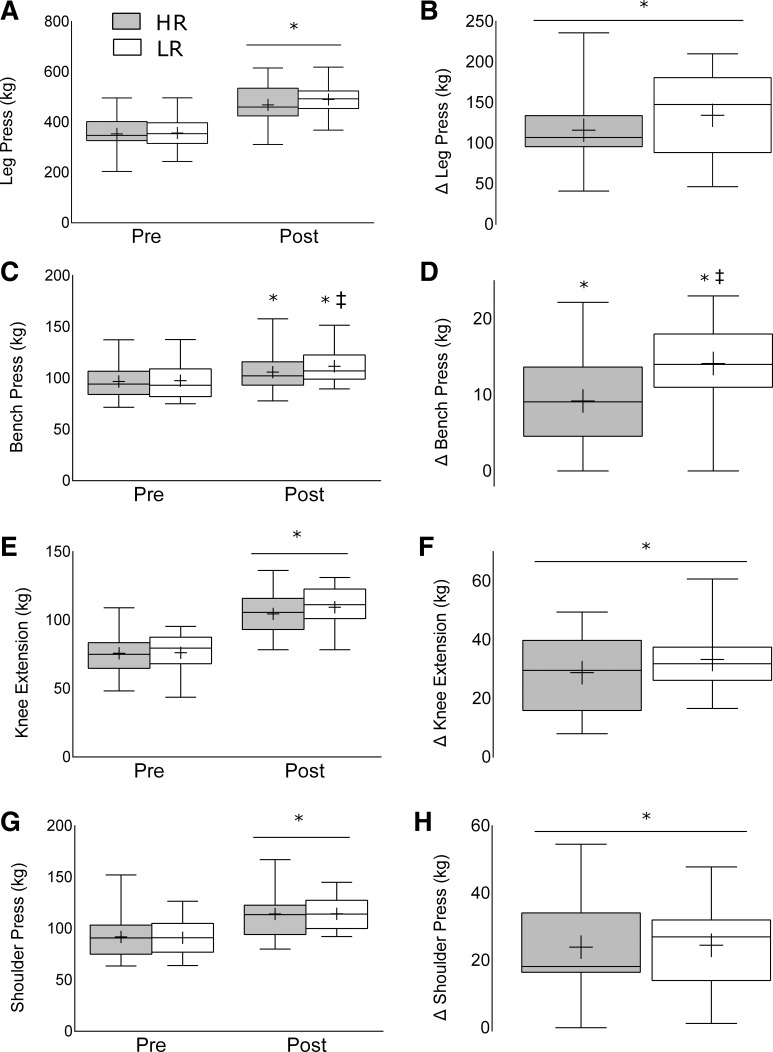
Strength changes in the high-repetition (HR) and low-repetition (LR) groups following 12 wk of resistance training for the leg press absolute values (*A*) and change following training (*B*), bench press absolute values (*C*) and change following training (*D*), knee extension absolute values (*E*) and change following training (*F*), and shoulder press absolute values (*G*) and change following training (*H*). Values are presented as median (lines) with interquartile range (boxes) ± range (minimum and maximum), where + indicates mean. *Significantly different (*P* < 0.05) from baseline. ‡Significantly different (*P* < 0.05) between HR and LR.

All exercises passed normality assessed by the Kolmogorov-Smirnov test (*P* > 0.05) with the exception of preintervention LP (*P* = 0.03) and BP (*P* = 0.01); however, assessment of histogram and probability-probability (P-P) plots revealed no kurtosis or skewness. Levene's test revealed no significance for any variable (*P* > 0.05). Maximum isotonic strength (using pooled means) increased for LP [355 ± 10 to 480 ± 11 kg; *F*(1,48) = 249.77, *P* < 0.001], KE [76 ± 2 to 107 ± 2 kg; *F*(1,47) = 216.91, *P* < 0.001], SP [91 ± 3 to 112 ± 12 kg; *F*(1,46) = 113.83, *P* < 0.001], and BP [97 ± 3 to 109 ± 3 kg; *F*(1,47) = 152.07, *P* < 0.001; Fig. 4] following the intervention. There were no group by time differences for LP, KE, or SP; however, the change in BP was greater in the LR group (14 ± 1 kg) than in the HR group [9 ± 1 kg; *F*(1,47) = 6.75, *P* = 0.012; Fig. 4, *C* and *D*]. Independent *t*-tests on the absolute change also revealed no significant difference between groups for LP [*t*(47) = −0.1, *P* > 0.05, M = −2.55, 95% CI (−53, 48)], KE [*t*(47) = −1.47, *P* > 0.05, M = −6.03, 95% CI (−14, 2)], and SP [*t*(47) = 0.55, *P* > 0.05, M = 4.3, 95% CI (−11, 19)]; however, as the ANOVA results showed, there was a significant difference between group difference for BP [*t*(47) = −2.6, *P* < 0.05, M = −4.9, 95% CI (−8.7, −1.1)].

#### Resistance-training volume.

Average volume per session was significantly lower in the LR group (14,805 ± 592 kg) than in the HR group (23,969 ± 901 kg; *P* < 0.001).

#### Hormone concentrations.

**Fig. 5.**
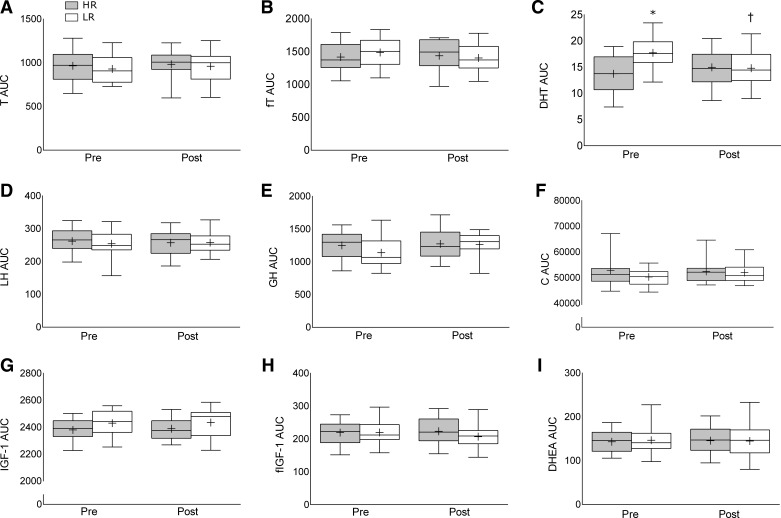
Acute postexercise area under the curve (AUC) preintervention and postintervention for testosterone (T; *A*), free testosterone (fT; *B*), dihydrotestosterone (DHT; *C*), luteinizing hormone (LH; *D*), growth hormone (GH; *E*), cortisol (C; *F*), insulin-like growth factor 1 (IGF-1; *G*), free IGF-1 (fIGF-1; *H*), and dehydroepiandrosterone (DHEA; *I*). Values are presented as median (lines) with interquartile range (boxes) ± range (minimum and maximum), where + indicates mean. HR, high-repetition group (20–25 repetitions per set); LR, low-repetition group (8–12 repetitions per set). *Significantly different (*P* < 0.05) from HR. †Significant group by time effect (*P* < 0.05).

Kolmogorov-Smirnov tests showed normality for all postexercise hormone AUCs (*P* > 0.05) with the exception of preintervention and postintervention cortisol (*P* < 0.001); however, assessment of histogram and P-P plots revealed little to no kurtosis or skewness. Levene's test revealed that preintervention lactate (*P* = 0.03), preintervention cortisol (*P* = 0.03), and postintervention lactate (*P* = 0.01) were significant. The hormone concentrations were not “corrected” for blood volume shifts, which have a negligible impact on the results, as we propose that the “uncorrected” concentrations are what the target tissues (i.e., muscle) would be exposed to in vivo. Every blood outcome (T, fT, DHT, DHEA, cortisol, IGF-1, fIGF-1, GH, LH, and lactate) increased as a result of the acute exercise bout (*P* < 0.001). There was a group difference preintervention for the postexercise AUC of DHT [HR, 13.6 ± 0.7; LR, 17.7 ± 0.7 ng·ml^−1^·min^−1^] with a group by time effect [HR, 1.2 ± 1; LR, −2.9 ± 0.8 ng·ml^−1^·min^−1^, *P* = 0.003] such that the postexercise AUC for DHT was similar between groups postintervention (Fig. 5). There were no other group, time, or group by time differences for any postexercise hormonal AUC.

#### Correlations.

There were weak to moderate correlations for a variety of hormones though the change in type II CSA with preintervention (*r* = −0.34, *P* = 0.02) and postintervention (*r* = −0.31, *P* = 0.04) cortisol, the change in LP with preintervention fIGF-1 (*r* = 0.40, *P* = 0.01), the change in SP with postintervention lactate (*r* = −0.36, *P* = 0.01), and the change in BP with preintervention LH (*r* = 0.43, *P* = 0.003) AUC were all significant (Table 2). No other hormone at any time point was significantly correlated with the change in hypertrophy or strength.

**Table 2.** Pearson correlation coefficients for the postexercise hormonal area under the curve preintervention and postintervention and measures of muscle hypertrophy and strength

	Postexercise AUC													
	T		fT		DHT		IGF-1		fIGF-1		GH		Cortisol	
	Pre	Post	Pre	Post	Pre	Post	Pre	Post	Pre	Post	Pre	Post	Pre	Post
Δ type I CSA	0.26	0.1	0.29	0.07	−0.1	0.13	0.06	0.17	−0.16	−0.03	−0.1	−0.28	−0.06	−0.07
Δ type II CSA	0.13	0.02	0.18	0.20	0.02	0.06	0.16	−0.02	0.02	−0.05	−0.2	−0.21	−0.34*	−0.3*
Δ LBM	−0.01	−0.02	0.08	−0.12	0.22	−0.26	0.15	0.11	0.25	−0.04	0.19	−0.01	0.05	0.26
Δ LP	0.26	0.1	0.02	0.10	0.06	−0.06	−0.2	−0.05	0.4*	−0.23	0.04	−0.12	−0.16	0.07
Δ BP	−0.12	0.23	−0.1	−0.11	0.14	0.1	−0.1	0.01	0.12	−0.09	−0.3	−0.15	−0.22	0.01

Change (Δ) in type I muscle fiber cross-sectional area (CSA), type II muscle fiber CSA, lean body mass (LBM), leg press (LP), and bench press (BP). The preexercise and postexercise hormone areas under the curve (AUCs, 60 min, see methods for details) are reported. Pre, preintervention; post, postintervention; T, total testosterone; fT, free testosterone; DHT, dihydrotestosterone; IGF-1, insulin growth-like factor 1; fIGF-1, free IGF-1; GH, growth hormone.

* Significantly correlated (*P* < 0.05).

## DISCUSSION

Twelve weeks of supervised, higher- and lower-load per repetition RT programs were similarly effective at inducing skeletal muscle hypertrophy in resistance-trained participants when RT was performed to volitional failure. Additionally, when participants were tested periodically for maximal strength (i.e., essentially being allowed to practice their 1RM), the increases in muscular strength were not significantly different between groups. The exception was bench press 1RM, which increased to a greater extent in the LR group. Additionally, postexercise levels of circulating hormones did not change as a result of the RT intervention and were unrelated to changes in muscle mass and strength.

The amount of mass lifted per repetition (referred to here as load) is not a primary determinant of changes in muscle protein synthesis (4) or hypertrophy (28) when resistance exercise is performed until volitional failure in untrained participants. Mitchell et al. (28) demonstrated greater gains in muscle mass than in the present study following 10 wk of RT in untrained participants who performed only knee extension thrice weekly [i.e., Mitchell et al. (28) vs. present study: type I CSA, ∼23 vs. ∼12%; type II CSA, ∼19 vs. 16%]. The attenuated gains in muscle size in the present study vs. those seen by Mitchell et al. (28) are congruent with previous literature showing a blunted training response in resistance-trained individuals, who would presumably have less capacity for adaptation since they are regularly exposed to the stimulus of RT (17, 42). Taken together with previous data (4, 28), the findings of the present study, along with a recent metaanalysis (35), do not support the assertion that higher-load RT is a prerequisite to maximize RT-induced muscle hypertrophy especially when lower-load exercises are performed to volitional failure.

Few studies have addressed the effect of load with hypertrophy and strength as main outcomes when the exercise sessions are not volume-matched (20, 28). Indeed, in a volume-matched situation, low-repetition (high load) RT appears to provide a greater stimulus for hypertrophy and strength gains (5, 15, 41); however, it is obvious that when performing RT with lighter loads, a greater lifting volume (repetitions × load) is needed to reach volitional failure. In the present study, which had participants perform RT until volitional failure, average session volume performed in the LR group was only ∼62% of that performed by the HR group. We hypothesize that the increased volume performed by the HR group allowed them to reach volitional failure, which led to the similar adaptations seen in the LR group, a finding consistent with previous studies (4, 28, 33). Alternatively viewed, performance of a LR set at 80% of 1RM and a HR set performed at 40% of 1RM would result, at volitional failure, in the LR set having lost only ∼20% of their force-generating capacity and the HR group having lost ∼60% of their force-generating capacity. To be clear, it is apparent that a HR group would have to perform more repetitions (thus more volume) and lose more of their force-generating capacity (fatigue) to reach volitional failure on any given set. While the mechanisms underlying fatigue may be different between groups (11, 22), at volitional failure the size principle would dictate that larger motor units have been recruited in an attempt to sustain the required force (14, 26). There have been recent claims that greater EMG amplitude seen with a higher- vs. a lower-load condition is equivalent to greater motor unit drive and thus greater potential for hypertrophy (21); however, such a premise is fundamentally incorrect as has been pointed out (45, 46). The current data, along with previous work (28, 35), are direct proof that hypertrophy and strength gains are not a function of the load lifted and directly contradict the assertion that acute EMG recordings predict hypertrophic potential (21). Instead, we propose that exercising until volitional failure with adequate volume and load (between 30–90% 1RM) will sufficiently activate muscle motor units, which drives skeletal muscle hypertrophy.

Studies that have used volume-matched groups often have participants lift in a lower-repetition (higher load) condition to volitional failure to determine the volume that the higher-repetition (lower load) group will match (15, 41). This scenario would, we argue, not allow the high-repetition group to perform their RT to volitional failure and would result in an inferior stimulus. For example, Holm et al. (15) examined untrained young men performing volume-matched unilateral RT and found that low-repetition RT resulted in a significantly greater increase in muscle CSA (measured via magnetic resonance imaging) compared with the high-repetition RT (7.6 vs. 2.6%, respectively). Indeed, work from our group using a similar model indicates that a higher-repetition, lower-load group volume-matched to a lower-repetition, higher-load group produces a substantially inferior muscle protein synthesis response (4). In contrast, however, lower loads, when lifted to volitional failure (i.e., using a greater volume than the higher-load condition), results in a similar stimulation of muscle protein synthesis (4) and equivalent hypertrophy (28). Even if different RT programs are manipulated to have participants exercise until volitional failure and be volume-matched (e.g., more sets) (34), it remains apparent that the similar adaptations are a result of the resistance exercise being performed until volitional failure. Thus, in the current protocol, our participants performed their RT, regardless of group assignment, to volitional failure. As mentioned previously, allowing the HR group to perform more volume, resulting in volitional failure, there was fatigue that would have driven motor unit recruitment (4, 28) and therefore hypertrophy of the muscle fibers innervated by both large and small motor units (28, 29).

Following the 12-wk intervention, there were similar increases in muscular strength between groups. Specifically, both HR and LR increased LP, KE, and SP 1RM with no differences between groups. However, while both groups increased BP 1RM, the increase was greater in the LR group compared with the HR group (15 vs. 9%; Fig. 4, *C* and *D*). Notably, others have also found similar increases in 1RM in healthy untrained (15) and trained (33) men performing either low- or high-load RT. It is evident that current literature supports the use of both low-repetition (high load) (1, 20, 41) and high-repetition (low load) (5, 28, 44) RT to induce increases in maximal strength. Our results support the concept that maximal strength increases can be achieved with the use of either low or high loads, so long as there is periodic practice of lifting with heavier loads, whereas the disparity in BP 1RM changes remain in agreement with literature supporting the use of high loads with a low repetition range. We have previously reported greater increases in isotonic 1RM when performing RT with high loads (80% 1RM) than low loads (30% 1RM); however, when strength was evaluated with an unpracticed test, a 5-s isometric maximum voluntary contraction using a dynamometer, there was no difference between groups (28). Indeed, strength is a product of muscle mass (23), neural adaptation (7, 32), and “practice” of the desired outcome. Though there is no apparent advantage of lifting with different loads on changes in muscle mass, there is undoubtedly a neuromuscular advantage to lifting heavier loads if the primary outcome is performing a 1RM test (28). Conversely, it appears that periodic practice of the chosen strength outcome (e.g., 1RM) is effective at eliminating the majority of any posttraining difference.

A further purpose of the current study was to investigate the effects of novel (DHT, DHEA, and LH) and canonical (IGF-1, GH, and T) postexercise, circulating hormones that have been hypothesized to provide an anabolic stimulus [for reviews, see Kraemer and Ratamess (19) and Vingren et al. (47)]. An acute bout of exercise induces a significant but transient systemic rise in a variety of hormones and metabolites (19). It has been previously reported that the postexercise hormonal environment does not contribute to the resistance exercise-induced muscle protein synthetic response (51) or hypertrophy following RT (50). Despite women having ∼15- and 45-fold lower resting and postexercise systemic T concentrations, respectively, men and women experience similar magnitudes of myofibrillar protein synthesis in response to the same RT stimulus (49). West and Phillips (52) concluded that anabolic hormones such as GH, IGF-1, and T have little to no correlation with changes in hypertrophy and strength as a result of a 12-wk RT intervention. The present study adds to these results by comparing the hormonal response to different (high and low load) RT regimens in resistance-trained persons. We observed no correlations, at any time point, between the postexercise AUC for T, GH, and IGF-1 and changes in muscle mass and strength. Last, the postexercise concentrations of any of the aforementioned hormones are not even moderately (*r* > 0.45) relevant indicators of RT-induced changes in muscle mass and strength in resistance-trained men (Table 2) and do not change as a result of RT (Fig. 5). We acknowledge that the acute exercise trial was conducted in the fasted state, which may limit the direct applicability of these data to the applied setting; however, when subjects were fed, we have also not observed relationships between hormones and hypertrophy (52).

It is important to acknowledge that our repetition ranges and loads were chosen to match previous study “intensities” (4, 5, 15, 28, 43, 44) and replicate those of current guidelines set forth by the American College of Sports Medicine (31) and National Strength and Conditioning Association (12). As mentioned before (28) and in a recent review (29), we propose that muscle hypertrophy is fundamentally driven by motor unit activation. The current data demonstrate that performing RT with high and low repetitions (using low and high loads, respectively) to volitional failure provides a similar and sufficient stimulus, though neither are necessary, for hypertrophy or strength. In conjunction with previous data (28), it appears that if 1RM strength is the primary goal, performing the to-be-tested exercise with heavier loads, either consistently and/or periodically, may be required for optimal improvement. Thus lifting heavier and lighter loads should not be mutually exclusive in terms of promoting RT adaptations, but as training “zones” that could easily be used in RT programs without the expectation that strength or muscle mass gains would be significantly compromised, though we acknowledge that training paradigms should be tailored to the individual's goals and preferences.

In conclusion, high- and low-repetition (low and high load, respectively) training paradigms elicit a comparable stimulus for the accretion of skeletal muscle mass when resistance exercise is performed until volitional failure. The current findings taken together with previous reports (1, 20, 28) show that these effects are not contingent upon training status or study design. Increases in lean body mass, as an indirect measure of muscle mass, and muscle fiber CSA, a direct measure of muscle area, occurred in both LR and HR groups with no differences between groups. There was a significant increase in 1RM strength for the leg press, knee extension, and shoulder press exercises, again with no differences between groups. While 1RM bench press increased in both groups, it increased to a greater extent in the LR group. We speculate that because the participants in the HR group performed greater volume, they were able to exercise until volitional failure, which allowed for maximal activation of their motor units and ultimately led to the similar increases in muscle strength and hypertrophy seen in the LR group. In agreement with previous studies (50–52) it is clear that the postexercise increases in systemic hormone concentrations are unrelated to changes in muscle hypertrophy or strength.

## GRANTS

The project was supported by an operating grant to S. M. Phillips from the Natural Science and Engineering Research Council of Canada. S. M. Phillips gratefully acknowledges the support of the Canada Research Chairs program during the completion of this work.

## DISCLOSURES

No conflicts of interest, financial or otherwise, are declared by the author(s).

## AUTHOR CONTRIBUTIONS

R.W.M., S.Y.O., C.M., and S.M.P. conception and design of research; R.W.M., S.Y.O., C.G.W., N.M., C.M., J.Q., S.K.B., and S.M.P. performed experiments; R.W.M., S.Y.O., C.G.W., N.M., C.M., B.L.B., and S.M.P. analyzed data; R.W.M., S.Y.O., C.G.W., N.M., C.M., S.K.B., and S.M.P. interpreted results of experiments; R.W.M., S.Y.O., C.M., J.Q., and S.M.P. prepared figures; R.W.M., S.Y.O., C.M., S.K.B., and S.M.P. drafted manuscript; R.W.M., S.Y.O., C.G.W., N.M., C.M., J.Q., S.K.B., and S.M.P. edited and revised manuscript; R.W.M., S.Y.O., C.G.W., N.M., C.M., J.Q., B.L.B., S.K.B., and S.M.P. approved final version of manuscript.
